# Dipeptide of ψ-GSH Inhibits Oxidative Stress and Neuroinflammation in an Alzheimer’s Disease Mouse Model

**DOI:** 10.3390/antiox11061075

**Published:** 2022-05-28

**Authors:** Abbas Raza, Wei Xie, Kwan-Hyun Kim, Venkateshwara Rao Dronamraju, Jessica Williams, Robert Vince, Swati S. More

**Affiliations:** Center for Drug design, College of Pharmacy, University of Minnesota, Minneapolis, MN 55455, USA; razax003@umn.edu (A.R.); wxie@umn.edu (W.X.); kwanhyun@gmail.com (K.-H.K.); drona006@umn.edu (V.R.D.); kell0586@umn.edu (J.W.); vince001@umn.edu (R.V.)

**Keywords:** Alzheimer’s disease, antioxidant, glutathione, precursor, ψ-GSH, oxidative stress, neuroinflammation

## Abstract

Supplementation of glutathione (GSH) levels through varying formulations or precursors has thus far appeared to be a tenable strategy to ameliorate disease-associated oxidative stress. Metabolic liability of GSH and its precursors, i.e., hydrolysis by the ubiquitous γ-glutamyl transpeptidase (γ-GT), has limited successful clinical translation due to poor bioavailability. We addressed this problem through the design of γ-GT-resistant GSH analogue, ψ-GSH, which successfully substituted in GSH-dependent enzymatic systems and also offered promise as a therapeutic for Alzheimer’s disease (AD). With the aim to improve its bioavailability, we studied the utility of a ψ-GSH precursor, dipeptide **2**, as a potential AD therapeutic. Compound **2** retains the γ-GT stable ureide linkage and the thiol group for antioxidant property. By engaging glutathione synthetase, compound **2** was able to generate ψ-GSH in vivo. It was found to be a modest cofactor of glutathione peroxidase and prevented cytotoxicity of Aβ^1–42^-aggregates in vitro. Studies of compound **2** in an acute AD model generated by intracerebroventricular injection of Aβ^1–42^ showed cognitive benefits, which were augmented by its combination with glycine along with mitigation of oxidative stress and inflammatory pathology. Collectively, these results support further optimization and evaluation of ψ-GSH dipeptide as a potential therapeutic in transgenic AD models.

## 1. Introduction

The increase in overall life expectancy now highlights the gravity of Alzheimer’s disease (AD) and related dementias in the elderly. All these disorders are primarily characterized by the initial impairment in cognitive function. Typical hallmarks of Alzheimer’s disease (AD) encompass extracellular deposition of amyloid-β plaques and intraneuronal aggregation of microtubule-associated protein tau, both of which are intricately linked with increased oxidative stress and neuronal inflammation [1,2]. Oxidative stress is evident from reduced levels of cellular antioxidant glutathione (GSH) [3], peroxidation of lipid membranes [4], protein carbonylation by reactive oxygen species (ROS), and oxidative damage to nucleic acids [5]. Senile Aβ-plaques are associated with activated microglia and astrocytes along with upregulation of proinflammatory cytokines, leading to neuronal dysfunction and ultimately death [6]. Although seen frequently in aging and other neurological disorders and not specific to AD, oxidative stress and inflammation are considered important biological alterations of AD pathology. Increasing evidence also indicates involvement of oxidative mechanisms toward pathogenesis of AD [7]. Due to its higher energy demand and oxygen consumption, brain tissue is especially susceptible to such oxidative damage. This is partly due to damage to the cellular energy production machinery, the mitochondria, resulting in extensive ROS generation [8]. These processes display a causal link between neuronal damage and cognitive dysfunction in many aging, experimental AD models and elderly individuals [9,10]. The findings have spurred the development of biomarkers and therapeutics for effective management of the disease [11]. General antioxidant strategies have attained success in numerous preclinical studies; however, determination of clinical utility of antioxidants would require further investigations [12]. Revisions to general antioxidant approaches to include development of multipronged antioxidants affecting multiple mechanisms of oxidative damage would be an ideal next step.

Decrease in the level of GSH is observed during AD pathogenesis and progression. Measurement of brain GSH levels using magnetic resonance spectroscopy [13] and recently blood GSH levels [14] have offered promise as diagnostic markers for early stages of AD. Efforts have also been made to supplement endogenous GSH stores by itself or its precursors [15]. However, the γ-glutamylcysteinyl amide bond in GSH is cleaved readily by a ubiquitous enzyme, γ-glutamyl transpeptidase (γ-GT). Such metabolic liability has limited the bioavailability of GSH and further thwarted clinical translation in this area [16]. Amongst the GSH precursors that have also been investigated, of particular note is the dipeptide, γ-glutamylcysteine. Endogenous synthesis of γ-glutamylcysteine by γ-glutamylcysteinyl ligase is considered the rate-limiting step in GSH biosynthesis, and thus, provision of this crucial dipeptide was deemed as a viable strategy to circumvent the regulation of GSH synthesis [17]. This dipeptide was able to mitigate oxidative stress and neuroinflammation caused by oligomers of Aβ^1–40^ in human astrocytic cultures and in a murine model of AD [18,19]. Importantly, other than being an antioxidant, the γ-glutamylcysteine dipeptide could also function as a cofactor of glutathione peroxidase-1 (GPx) enzyme, attenuating mitochondrial ROS [20]. However, this dipeptide and other precursors of GSH still retained the metabolic instability around the γ-glutamylcysteinyl bond.

**Figure 1:**
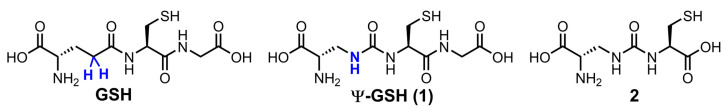
Chemical structures of ψ-GSH and the dipeptide of ψ-GSH.

We have addressed this shortcoming of GSH analogues by design of compound ψ-GSH, which replaces the labile amide bond with a γ-GT-resistant ureide linkage (Figure 1) [21]. When tested in enzymatic and cellular models, ψ-GSH substituted for GSH in the GSH-dependent enzymatic reactions utilized GSH transport mechanisms to cross the blood–brain barrier and detoxified cellular oxidative stress [21]. We have demonstrated efficacy of this compound in AD transgenic mice APP/PS1 in a preventative [22] and therapeutic [23] treatment regimens. Mechanistic investigations highlighted restoration of glyoxalase enzyme activity by ψ-GSH as a significant contributor to the latter’s neuroprotective activity. This enzyme system neutralizes toxic reactive dicarbonyls such as methylglyoxal, which are partly responsible for aggregation of Aβ^1–42^, to relatively non-toxic D-lactate using ψ-GSH as a cofactor in place of GSH [21,24]. Limited oral bioavailability is one of the shortcomings of ψ-GSH that needed our attention. One strategy to improve oral availability was to consider various precursors of ψ-GSH that would generate the tripeptide in situ. Given the success of the γ-glutamylcysteinyl dipeptide of GSH, we decided to evaluate the corresponding dipeptide of ψ-GSH, compound **2**, in cellular and mouse models of AD (Figure 1). The ψ-GSH dipeptide was expected to retain the antioxidant properties of parent compound ψ-GSH, offered by the thiol functionality present in both molecules and generate the tripeptide ψ-GSH in situ. Additionally, such a dipeptide, similar to GSH dipeptide, has the potential to restore mitochondrial glutathione peroxidase function, further broadening the implications of this approach.

This study reports for the first time the synthesis of ψ-GSH dipeptide, its antioxidant potency, and efficacy against Aβ^1–42^-toxicity in cell culture and in an AD-like mouse model generated by intracerebroventricular injection of Aβ^1–42^. We also evaluated this compound in glutathione peroxidase enzymatic assay and examined its pharmacokinetic conversion to the tripeptide ψ-GSH. The results of this study form a rational basis for further preclinical explorations of ψ-GSH dipeptide as an effective therapeutic agent against AD.

## 2. Materials and Methods

### 2.1. Chemistry, General Procedures

Nuclear Magnetic Resonance spectra were recorded on a Varian XL 400 MHz instrument. All ^1^H NMR shifts are reported in units, parts per million (ppm) and coupling constants were reported in Hz. Flash chromatography was performed using silica gel (100–200) with indicated mobile phase. Mass analyses were performed on an Agilent LC-TOF 6310 mass spectrometer equipped with either an ESI or APCI source.

Synthesis of *N*-(((S)-3-(*tert*-butoxy)-2-((*tert*-butoxycarbonyl)amino)-3-oxopropyl) carbamoyl)-*S*-trityl-*L*-cysteine (5). To a stirring solution of *tert*-butyl (*S*)-3-amino-2-((*tert*-butoxycarbonyl)amino)propanoate 4 (0.32 g, 1.1 mol) in CH_2_Cl_2_ (25 mL) under inert atmosphere were added *N*-methyl morpholine (0.3 mL, 2.69 mol) and *N*,*N*′-dicyclohexylcarbodiimide (0.21 g 1.1 mmol) at 0 °C and stirred at the same temperature for 30 min. To this were added *S*-tritylcysteine (0.43 g, 1.1 mmol) and *N*-methyl morpholine (0.3 mL, 2.69 mol) at 0 °C. The reaction mixture was gradually warmed to RT and stirred for 16 h. The reaction was monitored by TLC; after completion of the reaction, the reaction mixture was quenched with ice-cold water (10 mL) and the precipitated solid was filtered and dried under vacuum to obtain the crude. The crude was purified through silica gel column chromatography using 25–30% EtOAc/heptanes and further purified by precipitation using methyl *tert* butyl ether: *n*-pentane (1: 7, 1.5 L) to afford 5 (0.31 g, 40%) as white solid. ^1^H NMR (400 MHz, DMSO-*d*_6_): δ 7.37–7.17 (m, 15H), 7.10–6.97 (m, 1H), 6.59–6.42 (m, 1H), 6.34–6.23 (m, 1H), 4.22–4.04 (m, 1H), 3.92–3.79 (m, 1H), 3.44–3.32 (m, 1H), 3.21–3.09 (m, 1H), 2.47–2.39 (m, 2H), 1.40, 1.38 (s, 18H); LC-MS (Agilent 6310 Ion Trap): *m*/*z* 648.3 [M − H]; (column; Kinetex Evo (50 × 3 mm, 2.6 µm); RT 2.97 min. 2.5 mM Aq NH_4_OOCH + 5% ACN: ACN + 5% 2.5 mM Aq NH_4_OOCH; 0.8 mL/min).

Synthesis of (*S*)-2-amino-3-(3-((*R*)-1-carboxy-2-(tritylthio)ethyl)ureido)propanoic acid (6). To a pre-cooled 98% formic acid (10 mL) at 0 °C was added *N*-(((S)-3-(tert-butoxy)-2-((*tert*-butoxycarbonyl)amino)-3-oxopropyl)carbamoyl)-*S*-trityl-*L*-cysteine **5** (0.54 g, 0.83 mmol) under inert atmosphere and the reaction mixture was gradually warmed to 45 °C and stirred for 16 h. The reaction was monitored by LC-MS; after completion of the reaction, the volatiles were removed in vacuo at 30 °C (water bath temperature) to obtain the crude. The crude was triturated with diethylether (20 mL), CH_3_CN (25 mL) and dried in vacuo to afford **6** (200 mg, 49%) as an off-white solid. ^1^H NMR (400 MHz, DMSO-*d*_6_): δ 7.42–7.16 (m, 17H), 6.76–6.69 (m, 1H), 6.68–6.53 (m, 1H), 4.16–4.04 (m, 1H), 3.48–3.28 (m, 1H), 3.27–3.19 (m, 2H), 2.47–2.40 (m, 1H), 2.39–2.30 (m, 1H); LC-MS (Agilent 6310 Ion Trap): *m*/*z* 493.7 [M + H]; (column; Kinetex Evo C-18 (50 × 3 mm, 2.6 µm); RT 3.07 min. 2.5 mM Aq NH_4_OAc ACN; 0.8 mL/min).

Synthesis of (*S*)-2-amino-3-(3-((*R*)-1-carboxy-2-mercaptoethyl)ureido)propanoic acid TFA salt (2). To a stirring solution of (*S*)-2-amino-3-(3-((*R*)-1-carboxy-2-(tritylthio)ethyl)ureido)propanoic acid (**6**) (1.8 g, 3.65 mmol) under inert atmosphere in CH_2_Cl_2_ was added trifluoroacetic acid (35 mL) dropwise for 20 min at 0 °C, followed by addition of triethylsilane (3.5 mL) at 0 °C. The reaction mixture was gradually warmed to RT and stirred for 16 h. The reaction was monitored by LC-MS; after completion of the reaction, the volatiles were removed in vacuo at below 20 °C to obtain the crude. The crude was triturated with diethylether (50 mL) and dried under vacuum to obtain the solid. The obtained solid was dissolved in water (30 mL, Milli-Q), washed with EtOAc (80 mL). The aqueous layer was lyophilized to afford the compound (1 g, 75%) as an off-white fluffy solid. ^1^H NMR (400 MHz, D_2_O): δ 4.57–4.48 (m, 1H), 4.09–4.00 (m, 1H), 3.87–3.76 (m, 1H), 3.71–3.59 (m, 1H), 3.17–2.94 (m, 2H); LC-MS (Agilent 6310 Ion Trap): *m*/*z* 251.9 [M + H]^+^; (Column; Synergi Fusion RP (150 × 4.6 mm, 4.0 µm); RT 2.68 min. 0.1% Aq TFA; 1.0 mL/min). The NMR data and purity analysis of the final compound are included in the Supporting Information, Figure S1.

### 2.2. Antioxidant Activity by DPPH Assay

The antioxidant activity of ψ-GSH dipeptide was estimated by 1, 1- diphenyl-2-picryl hydrazyl (DPPH). Briefly, 0.1 mM solution of DPPH was prepared in 80% methanol. The stock solution of GSH, ψ-GSH dipeptide, or ascorbic acid was prepared at different concentrations (0.003, 0.01, 0.03, 0.1, 0.3, 1, 3, 10 mM). The assay was performed in a 96-well flat-bottom microplate with 180 µL of DPPH solution and 20 µL of sample in triplicate testing. The mixture was allowed to incubate in the dark at room temperature for 1.5 h. The absorbance at 515 nm was read using a Spectramax M5 reader. The percentage inhibition was calculated by using the following formula: (1 − A_S_/A_C_) × 100 where A_S_ was absorbance in presence of sample while A_C_ was that of control.

### 2.3. Glutathione Peroxidase Assay

Measurement of glutathione peroxidase activity was conducted in a coupled enzymatic reaction with glutathione reductase. Consumption of NADPH for reduction of oxidized GSH was used as a measure of the enzymatic activity. Briefly, various concentrations of GSH or ψ-GSH dipeptide were incubated with glutathione peroxidase (1.25 mU/mL), glutathione reductase (1.25 mU/mL), and NADPH (25 μM) in assay buffer (50 mM Tris pH 8.0, 0.5 mM EDTA). The reaction was initiated by addition of *tert*-butyl hydroperoxide (0.15 mM). All indicated concentrations are final concentrations in the reaction mixture. The absorbance at 340 nm was read using a Spectramax M5 reader. A decrease in absorbance was used as an indicator of NADPH consumption and enzyme activity.

### 2.4. Cellular Toxicity of Aβ^1–42^ in SH-SY5Y Cells

The human neuroblastoma cell line, SH-SY-5Y, was acquired from Sigma-Aldrich (St. Louis, MO). Cell culture medium MEM:F12 (1:1) medium supplemented with 15% FBS, 100 units/mL penicillin, and 100 units/mL streptomycin, 1% glutamax-1 and 1% NEAA (nonessential amino acid) was used for maintenance of cells. Cells were grown at 37 °C in a humidified atmosphere with 5% CO_2_/95% air. SH-SY5Y cells were plated in 96-well plates at a density of 20,000 cells/well. After overnight incubation at 37 °C, the cells were pretreated with compounds for 24 h. Then, the compound-containing medium was replaced with fresh compound-containing medium or a mixture of compound and Aβ^1–42^ (20 µM) for an additional 24 h. At the end of this treatment period, CCK-8 solution was added to each well and incubated for additional 3 h at 37 °C before the optical density at 450 nm was read with a microplate reader. Results are presented as mean percent of cell growth with respect to control with the standard error of the mean.

### 2.5. Glutathione Synthetase Assay

Measurement of glutathione synthetase activity was conducted in 200 μL of assay buffer (20 mM MgCl_2_, 2 mM EDTA, 50 mM KCl, 2.5 mM DTT, 5 mM glycine, and 10 mM ATP in 100 mM Tris pH 8.0) at 37 °C. The reaction was initiated by addition of 5 mM γ-Glu-Cys or compound **2** and 0.37 μM GSS. Incubations were continued for 1 h and 10 μL aliquots were removed at various time points, followed by quenching with 90 μL of 10% sulfosalicylic acid. Samples were analyzed by LC-MS/MS for the levels of GSH or ψ-GSH tripeptides.

### 2.6. Pharmacokinetic Analysis of ψ-GSH Dipeptide

Eight-week old CD-1 male mice were distributed into two groups (*n* = 5) and administered with 250 mg/kg of ψ-GSH, or dipeptide of ψ-GSH (**2**) in saline intraperitoneally. Blood samples were collected at 0, 5, 15, 30, 60, 90, 120, 240, and 360 min after the drug administration through saphenous vein bleeding into EDTA-K2 coated tubes. The sample was immediately centrifuged at 3000 rpm for 10 min at 4 °C. The plasma was transferred into a new tube and mixed with an equal volume of 3% sulfosalicylic acid in water. After centrifugation, the supernatants were injected into LC-MS/MS for analysis.

LC-MS/MS was performed on an Agilent 1260 HPLC (high–performance liquid chromatography) device coupled with an AB Sciex QTRAP 5500 mass spectrometer. The samples were separated using a Thermo Aquasil C18 column (150 × 2.1 mm, 3 µm) with a mobile phase of 0.1% formic acid in water (mobile phase A) and 0.1% formic acid in acetonitrile (mobile phase B) at a flow rate of 0.3 mL/min. The analytes were eluted with a smooth gradient as follows: from 0 to 0.5 min, 0–5% B (*v*/*v*); from 0.5 to 2.3 min, 5–10% B (*v*/*v*); from 2.3 to 2.5 min, 10–50% B (*v*/*v*); from 2.5 to 3.3 min, 50% B (*v*/*v*); from 3.3 to 3.5 min, 50–0% B (*v*/*v*); from 3.5 to 7.5 min, 0% B (*v*/*v*). Only the desired fraction of eluates (1.7–2.5 min) was diverted into the mass spectrometer for analysis. Samples were analyzed with an electrospray ionization source operated in the positive mode. Source and gas parameters were optimized, set to the following: curtain gas, 25 psi; CAD gas, medium; ion spray voltage, 5000 V; temperature, 700 °C; gas 1, 60 psi; gas 2, 50 psi. Multiple reaction monitoring (MRM) was conducted by monitoring the following transition: m/z 309.1→105 (ψ-GSH); 252.1→105 (ψ-GSH Dipeptide).

### 2.7. Efficacy Evaluation in Intracerebroventricularly Injected Aβ^1–42^ Mouse Model

The amyloid-β peptide solution was prepared for administration to C57BL/6 mice as described previously [25]. Briefly, Aβ^1–42^ treated with HFIP, reconstituted with DMSO to 1 mM stock solution, and then diluted with DPBS to a stock solution of 100 µM solution. After incubation of the Aβ^1–42^ solution incubated at 37 °C for 3 days, Aβ^1–42^ oligomers solution was intracerebroventricularly (i.c.v) injected to mice as described previously (1 nmol/mouse) [26]. The same volume of methylene blue solution was used to inject a group of mice to confirm the correctness of the intracerebroventricular injection site.

Aβ^1–42^ oligomers solution injected C57BL/6 mice were randomly distributed into the following groups (*n* = 7–9 per group): (1) the Aβ^1–42^ group (saline, p.o.), and (2) the Aβ^1–42^ + **1** group (500 mg/kg, p.o.). (4) the Aβ^1–42^ + **2** (500 mg/kg, p.o.). (5) the Aβ^1–42^ + **2** (250 mg/kg, i.p.) (6) the Aβ^1–42^ + **2** (500 mg/kg, i.p.). For the experiments where efficacy of the combination of compound **2** with glycine was tested, both compounds were administered intraperitoneally at 250 mg/kg dose.

The compound solutions were prepared with saline and were administered orally (p.o.) or intraperitoneally (i.p.) to mice at a dose of 500 mg/kg or 250 mg/kg once a day for 12 days. The spatial learning and memory of mice were assessed on days 10 and 11. After behavioral assessment, the animals were euthanized, gross dissection was performed, and tissues were collected for further analysis.

### 2.8. Spontaneous Alternation T-Maze Test

Cognitive function in this study was assessed through spontaneous alternation T-maze, following established protocols [25]. Prior to testing, all mice were allowed to acclimatize to the testing room for at least one hour. In the trial, the mouse was gently placed at the distal part of the start arm and allowed to freely explore the whole maze. Following the free exploration, when the mouse returned to the start arm, the guillotine door behind the subject was closed, and the mouse was kept in the start arm for 30 s confinements. Then, a central partition was gently installed, and the guillotine door was removed. The mouse was allowed to finish 15 free-choice trials. Mice with more than 30 min in 15 free-choice trials were excluded from further analysis. The percentage of alternation was calculated by the standard formula: (alternation/14 × 100). Three entries to the same arm was counted as one repetitive arm entry. The ratio of right to left side entry was used to evaluate the side preference.

### 2.9. Brain Tissue Preparation

Gross dissection was performed on each mouse after euthanasia. The mouse brain was collected. One hemisphere was rapidly frozen and stored at −80 °C for further biochemical analysis. The other hemisphere was post-fixed with a fresh 4% paraformaldehyde solution. After 24 h, the hemisphere brain was subsequently immersed into 20% sucrose in PBS until the tissue had sunk and then transferred to the 30% sucrose solution in PBS until sectioning.

### 2.10. Analysis of GSH and Protein Carbonyls in Mouse Brain

The oxidative stress markers, GSH, and protein carbonyls assays were performed using protocols described previously [25]. A GSH assay kit (Cayman Chemical, #703002, Ann Arbor, MI, USA) was used to measure the GSH levels in mouse brain homogenate. The assay is based on the reaction between DTNB (5,5′-dithiobis-2-nitrobenzoic acid) and GSH sulfhydryl group after an enzymatic recycling method through glutathione reductase. The formation of TNB was determined to estimate the GSH levels in the sample.

The protein carbonyls were determined using the protein carbonyls colorimetric kit (Cayman Chemical, #10005020, Ann Arbor, MI, USA). The amount of protein-hydrazone products was measured to quantify the protein carbonyl level after DNPH reaction with protein carbonyls in the brain homogenate. The values were then normalized to protein concentration in these samples.

### 2.11. Immunohistochemistry of Neuroinflammatory Markers

The cryopreserved brain tissues were cut into 8 μm frozen coronal sections using a cryostat (Leica, Wetzlar, Germany). After being air dried at ambient temperature for 30 min, the sections were fixed with cold methanol-acetone mixture (1:1) at −20 °C for 10 min. Antigen retrieval was performed with microwave heat-assisted antigen retrieval in citrate buffer (10 mM citrate buffer containing 0.05% Tween 20, pH 6.0) for 5 min. The section was stained with GFAP antibody (1:1000) overnight at 4 °C. Then, the anti-rabbit IgG HRP conjugated secondary antibody was applied for 1 h before the DAB kit was used for visualization, with counterstaining by Mayer’s hematoxylin solution. Expression of GFAP was quantitated by manual counting of GFAP-positive neurons and confirmation with stereological counting of the section, similar to the methods described in our previous publication [23].

## 3. Results

### 3.1. Synthesis of ψ-GSH Dipeptide

**Scheme 1:**
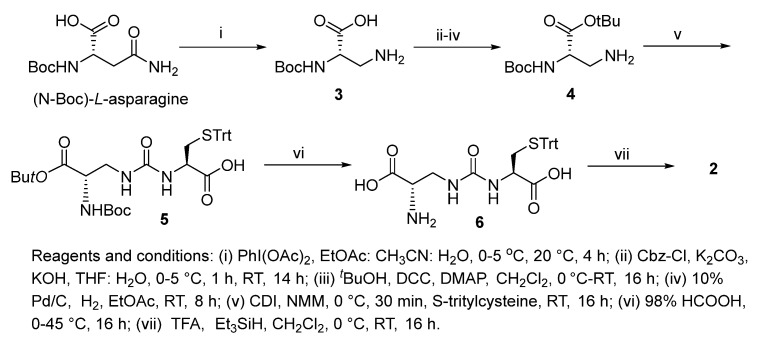
Synthesis of ψ-GSH dipeptide (**2**).

Increasing evidence indicates impaired glutathione biosynthesis in the normal aging and etiology of age-related disorders such as Alzheimer’s disease. The utility of direct GSH supplementation by oral and intraperitoneal routes is however limited due to its cleavage by intestinal γ-GT and rapid oxidation. With the incorporation of ureide linkage at the susceptible γ-glutamylcysteine amide, we have designed a γ-GT-resistant analogue of GSH that is able to substitute in GSH-dependent enzymatic systems. The efficacy of intraperitoneal ψ-GSH is evident from our studies in transgenic APP/PS1 Alzheimer’s mice. Oral administration of ψ-GSH was effective in rescuing hepatic damage caused by high-dose acetaminophen. The treatment of chronic aging diseases such as AD by oral ψ-GSH provided minimal benefit, requiring larger doses of the compound (manuscript under review). This shortcoming led us to evaluate several precursors and prodrugs of ψ-GSH for effective oral treatment of AD. This study describes the dipeptide precursor of ψ-GSH, our attempt at utilization, and biological evaluation of a ψ-GSH precursor. Synthesis of the dipeptide (**2**) utilized established methods in our lab for synthesis of the parent tripeptide (**1**, Scheme 1). The initial steps involved phenyliodine(III) diacetate (PIDA)-mediated Hofmann rearrangement of N-Boc-asparagine resulting in Boc-protected diaminopropanoic acid (**3**). Compound **3** was subjected to Cbz-protection of the primary amine, *t*-butyl esterification, followed by deprotection of the Cbz group resulting in unblocked diaminopropanoic acid (**4**). Formation of ureide linkage between **4** and *S*-tritylcysteine was conducted in the presence of carbonyldiimidazole (CDI), resulting in protected form of the dipeptide **5**. Sequential acidic deprotection of the trityl and *t*-butyl ester groups afforded the dipeptide of ψ-GSH (**2**).

### 3.2. ψ-GSH Dipeptide Retains the Antioxidant Property of the Tripeptide ψ-GSH

**Figure 2:**
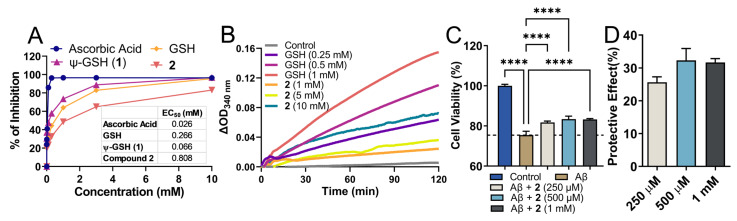
Antioxidant property of the dipeptide of ψ-GSH (**2**) and its ability to protect against Aβ^1–42^ cytotoxicity in SH-SY5Y cells. (**A**) Analysis of antioxidant property of compound **2** by DPPH assay. (**B**) Glutathione peroxidase enzymatic assay. Measurement of glutathione peroxidase activity was performed using the purified enzyme in the presence of GSH or **2** as cofactors. In this coupled enzymatic assay, consumption of NADPH by glutathione reductase to reduce oxidized glutathione or **2** was measured. Differences in the levels of NADPH measured as absorbance at 340 nm compared to time zero are plotted. (**C**) Cytotoxicity of Aβ^1–42^ in SH-SY5Y cells was mitigated by co-incubation with compound **2** in a dose-dependent manner. Plot (**D**) shows dose-dependent protective effect of **2** calculated in relation to Aβ-only group. Data are presented as the mean ± SEM of three independent experiments. Treatment groups were analyzed for statistical significance using one-way ANOVA with Tukey’s post-hoc test. **** *p* < 0.0001.

Being a structural mimetic of GSH and ψ-GSH, compound **2** is expected to display antioxidant properties. Such properties could be delivered by the dipeptide itself as well as by its conversion to tripeptide ψ-GSH in the biological system. The free thiol functionality present in these molecules is responsible for donating electrons for neutralization of ROS. We utilized a standard DPPH assay to evaluate free radical quenching potency of **2** by itself (Figure 2A). Co-incubation of the DPPH radical in the presence of compound **2** and reference compounds displayed loss of DPPH absorbance. Ascorbic acid was used as a control showed rapid quenching of DPPH (data collected at 30 min), while GSH and its analogs showed a relatively slower, sustained reaction (data collected after incubation for 1.5 h). In relation to GSH and ψ-GSH, compound **2** displayed slower reaction kinetics with DPPH. The concentration required for reduction of DPPH by 50% was calculated as EC_50_, for comparison of reactivity of these compounds. While ψ-GSH at a final concentration of 66 μM displayed 50% quenching of DPPH, much higher concentrations were required by GSH and **2**. The EC_50_ for GSH and **2** were 266 and 808 μM, respectively. This translated to glutathione equivalent antioxidant capacity (GEAC) of 4.04 and 0.33 for ψ-GSH and compound **2**, respectively. It is important to note, however, that the antioxidant property of **2** in biological systems will be an aggregate effect of the dipeptide and resulting tripeptide formed. In a cellular experiment to determine antioxidant potential of compound **2**, we were indeed able to see enhancement in its potency. ROS generated by peroxide treatment was quantitated using DCFDA fluorescent probe (Supporting Information, Figure S2). Increased intracellular ROS concentration resulting from H_2_O_2_ (500 μM) exposure was mitigated by compound **2** treatment (1 mM). Compared to direct incubation with DPPH radical, cellular enhancement in antioxidant activity of **2** could indicate its conversion to ψ-GSH leading to higher intracellular ROS quenching potential. Under similar conditions, GSH and ψ-GSH were also found to be efficacious in quenching cellular ROS in our previous study [21].

### 3.3. Glutathione Peroxidase Mediated Detoxification of Peroxides by ψ-GSH Dipeptide

In addition to direct quenching of the free radicals by the thiol group, the dipeptide could substitute for GSH in antioxidant enzyme reactions as a cofactor. Glutathione peroxidase is one such major enzyme that is involved in the detoxification of hydrogen peroxide and GSH acts as a cofactor. We sought to evaluate the potential of compound **2** to substitute for GSH in this enzymatic reaction (Figure 2B). Previous studies have demonstrated utilization of a range of thiol derivatives as cofactors by the peroxidase enzyme, although with lower efficiency [27]. The structural requirement for GSH analogs was deemed to be the sulfhydryl and glutamyl carboxylic acid groups [28] and both are conserved in this dipeptide of ψ-GSH, making it a likely candidate.

Enzymatic reactions with GPx and compound **2** were conducted in the presence of glutathione reductase which in turn reduced the oxidized form of **2** using NADPH. Consumption of NADPH was considered as an indicator of enzymatic activity. The data were plotted in relation to time zero and displayed the difference in absorbance at 340 nm. A dose-dependent increase in ΔOD was observed with GSH as a cofactor, while much higher concentrations of compound **2** were required to achieve measurable enzymatic rate. When tested as a cofactor, ψ-GSH (**1**) displayed cofactor efficiency equivalent to GSH in this assay (Supporting Information, Figure S3). Although lower efficiency was detected for the dipeptide **2**, it could be deemed as a poor cofactor of GPx.

### 3.4. Neurotoxic Effects of β-Amyloid 1–42 Are Ameliorated by the ψ-GSH Dipeptide

The main pathological hallmark of AD is amyloid plaques, which are composed of several Aβ species of variable lengths [29]. Aβ^1–42^ is the more aggregation-prone, fibrillogenic peptide that is formed by sequential proteolytic cleavage by secretases [30]. Aggregation of Aβ^1–42^, specifically soluble aggregates of Aβ^1–42^, are responsible for neuronal toxicity and ultimately death [31]. Oxidative stress plays an intricate part in formation and toxicity of Aβ^1–42^ and various antioxidant treatments have shown promise in mitigating such effects. We evaluated the potential of dipeptide **2** to protect against cytotoxicity of Aβ^1–42^ in SH-SY5Y cells (Figure 2C,D).

Incubation of SH-SY5Y cells with Aβ^1–42^ showed reduced cell viability after a 24 h incubation period. In contrast, treatment with compound **2** rescued cells against toxic effect of Aβ^1–42^ and attenuated cell viability in vitro after 24 h. Under similar experimental conditions, GSH and ψ-GSH were also found to be protective against Aβ^1–42^ cytotoxicity [21]. These data suggest that ψ-GSH dipeptide is able to cross cell membrane and increase the levels of GSH/ψ-GSH levels, which in turn attenuates intracellular oxidative stress and cytotoxicity of Aβ^1–42^ due to antioxidant effects of the compound itself and possibly by the resulting antioxidant tripeptides.

### 3.5. The ψ-GSH Dipeptide Acts as a Substrate of Glutathione Synthetase, Resulting in Formation of Tripeptide ψ-GSH

The biosynthesis of GSH involves two crucial steps, first by formation of γ-glutamylcysteinyl linkage by formation of γ-glutamyl cysteine, followed by addition of glycine by glutathione synthetase (GSS) resulting in the tripeptide GSH. Our rationale was to overcome the rate-limiting step of formation of γ-glutamyl amide bond by direct provision of the dipeptide. An added benefit of the ureide linkage is stability against γ-GT-mediated cleavage. Deemed as a precursor of ψ-GSH, dipeptide **2** is expected to utilize the GSS pathway to synthesize the active tripeptide ψ-GSH. We conducted an enzymatic assay of GSS to test this possibility (Table 1). When incubated in the presence of GSS, compound **2** indeed displayed formation of ψ-GSH. The concentration and time course of tripeptide formation by **2** were comparable to that of endogenous substrate γ-Glu-Cys under the same reaction conditions.

**Figure 3:**
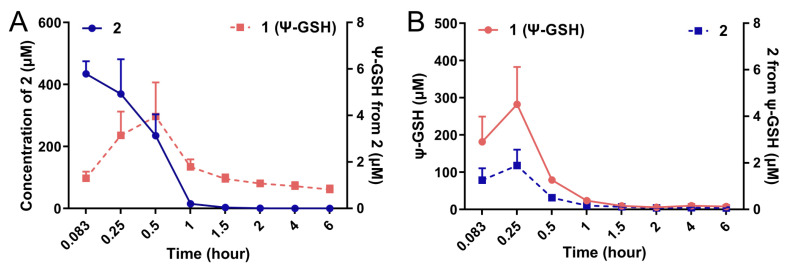
Conversion of compound **2** to the tripeptide ψ-GSH (**1**) after administration in mice. Compounds **1** and **2** (250 mg/kg) were injected in 8–10-week old CD1 mice by intraperitoneal route. Quantitation of ψ-GSH (**1**) formed after injection of **2** (**A**) and vice versa (**B**) was performed using LC-MS/MS analysis. Formation of tripeptide **1** from the dipeptide **2** was detected, although at much lower levels.

We attempted to measure the formation of ψ-GSH after administration of the dipeptide **2** in vivo in an intact animal after intraperitoneal administration. Figure 3 shows the plots of formation of ψ-GSH from **2** (Figure 3A) and formation of **2** after administration of ψ-GSH (Figure 3B) in CD1 mice. Compound **2** was indeed able to form ψ-GSH over the time course tested. Similarly, ψ-GSH underwent hydrolysis to **2** in the change time period. The extent of conversion between compound **2** and ψ-GSH was rather limited, with respective concentration of the metabolite about 100-fold lower than the parent compound. Short plasma half-life of these compounds discouraged further exploration of this phenomenon. When incubated with mouse brain homogenates, however, measurable formation of tripeptide ψ-GSH was detected (Supporting Information; Figure S4). The level of conversion was comparable to the concentrations produced by the endogenous substrate, γ-Glu-Cys. Additional metabolites that were detected include oxidized forms of the dipeptide **2** and tripeptide ψ-GSH. The ureide linkage was found to be stable toward proteolytic degradation within the timeframe of this experiment (6 h). Thus, this experiment indicates formation of the active tripeptide ψ-GSH as an alternative mode of protective mechanism by compound **2** against amyloid beta toxicity. It is likely that the modest inherent antioxidant capacity and GPx cofactor efficacy of **2** could also contribute toward the mechanism of action of this compound. The dosing schedule and brain accumulation would likely dictate contribution by these various factors.

### 3.6. Restoration of Cognitive Function in i.c.v. Aβ^1–42^ Injected Mice by Administration of Compound 2

To study the in vivo implications of compound **2** treatment against neuronal toxicity exhibited by Aβ^1–42^, we utilized an intracerebroventricularly (i.c.v.) injected Aβ^1–42^ model of AD. This model displays cognitive impairment and some of the major consequences of AD pathology such as oxidative stress and neuroinflammation [26]. Although it would be desirable to utilize transgenic mouse models of AD that closely replicate important clinical aspects of human AD, such studies are extensive and require treatment with compounds for longer duration. The i.c.v. Aβ^1–42^ model allows screening of potential drug candidates within the two-week treatment period, thus allowing identification of the most promising lead for further investigation in the transgenic models, greatly aiding drug development efforts. The solution of oligomeric Aβ^1–42^ is injected in cerebral ventricles that results in neuronal toxicity. This is in agreement with the currently accepted notion that the soluble aggregates of Aβ^1–42^ are the neurotoxic entities and contribute toward cognitive impairment observed in AD [32].

**Figure 4:**
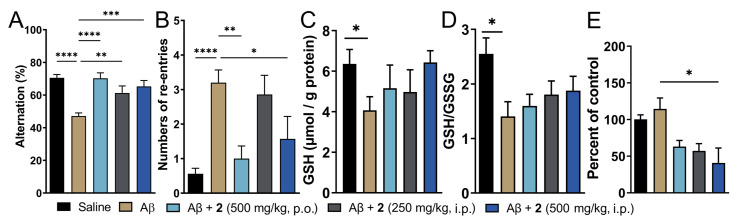
Treatment with ψ-GSH dipeptide (**2**) restored cognitive impairment induced by i.c.v. Aβ^1–42^. After initiation of compound treatment, i.c.v. injection of Aβ^1–42^ was performed on day 4 and followed by continued treatment with compound **2** for 8 additional days. The T-maze spontaneous alternation test was conducted on days 10 and 11 to assess cognitive function. (**A**) Reduced alternation behavior was observed in saline treated Aβ-injected mice, which was restored to levels comparable to vehicle control mice with oral and i.p. treatment of **2**. (**B**) Higher number of re-entries was evident in Aβ^1–42^-injected mice. Treatment with **2** (oral and high dose i.p.) showed reduction in repetitive arm entries. (**C**,**D**) Measurement of oxidative stress in the brain tissues was performed by analysis of reduced GSH and GSH/GSSG ratio. Compound-treated mice did not differ significantly from the vehicle-treated mice. (**E**) Quantitation of protein carbonyls by DNPH assay showed significant reduction in protein oxidation in the compound treated groups. Data are shown as the mean ± SEM. Statistical significance was assessed by a one-way ANOVA with Tukey’s post-hoc test (* *p* < 0.05, ** *p* < 0.01, *** *p* < 0.005, **** *p* < 0.001).

Treatment with compound **2** by i.p. and p.o. route was initiated three days before Aβ^1–42^ injection and continued for 8 additional days thereafter. Amongst the two doses of compound **2** administered intraperitoneally, the higher dose (500 mg/kg) displayed signs of toxicity as judged by more than 10% loss in body weight in 2 out of 8 mice tested (Supporting Information, Figure S5). The same dose given orally was, however, tolerated by all the animals in the treatment group. Cognitive function in these mice was assessed by T-maze spontaneous alternation test, which relies on the innate exploratory behavior of these mice to explore a novel environment (Figure 4). This protocol measures spatial working memory, which is influenced by dysfunction in various regions of the brain including hippocampus, forebrain, prefrontal cortex, or other parts of the brain. Compared to saline-injected mice, i.c.v. Aβ^1–42^-injected mice displayed reduced percentage alteration (70.54% for vehicle control vs. 47.14% for Aβ^1–42^-only group, respectively; Figure 4A). This was recapitulated in increased tendency to repetitive arm entries exhibited by these mice (0.56 for vehicle control vs. 3.2 for Aβ^1–42^-only group; Figure 4B). Both i.p. and orally treated groups of compound **2** displayed significant improvement in the cognitive behavior. The percent alternation and repetitive behavior was almost similar to the vehicle-treated controls. Oral treatment of **2** fared marginally better in restoration of cognitive function compared to the i.p. treated group. This could be explained by better oral safety profile of **2** and additionally, due to higher levels of ψ-GSH achieved after oral treatment of **2** due to hepatic expression of GSS enzyme. Higher abundance of GSS in the liver could potentially lead to higher conversion of **2** to the tripeptide ψ-GSH.

### 3.7. Improved Brain Redox Status and Mitigation of Neuroinflammation in the Brains of Mice Treated with Compound 2

Oxidative stress is evident in biochemical changes observed in the AD brain. Such changes are observed as protein and lipid oxidation, nucleotide damage, and ROS generation, which ultimately affect their biological functions accelerating the disease progression [5]. One of the consequences of oxidative stress is reduced levels of endogenous antioxidants such as GSH [14]. We expect that administration of compound **2** could affect the pool of cellular levels of reduced GSH, in addition to supply of bioactive ψ-GSH to neuronal cells. Quantitation of GSH levels in the i.c.v. Aβ^1–42^ treated mice showed significantly diminished levels of reduced GSH (Figure 4C). This correlated with significantly reduced redox status as judged by GSH/GSSG ratio (Figure 4D). These levels were affected by compound **2** treatment, with a trend toward increased GSH content and improved redox ratio.

**Figure 5:**
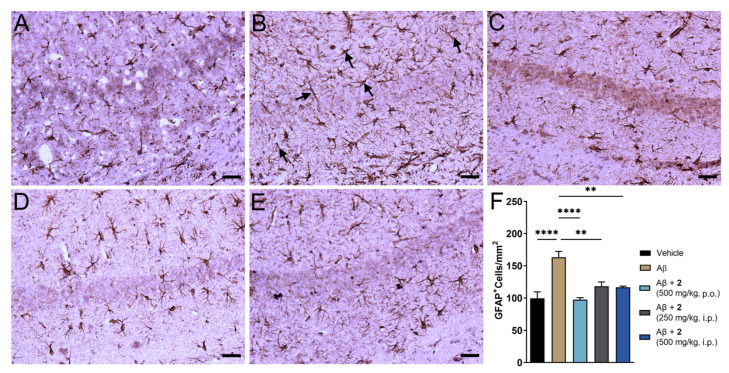
ψ-GSH dipeptide (**2**) treatment reduced reactive astrogliosis in i.c.v. Aβ^1–42^ injected mice. (**A**–**E**) Representative images of reactive astrocytes visualized by GFAP antibody in hippocampus region of various treatment groups: saline (**A**), Aβ-only (**B**), Aβ + **2** (500 mg/kg, p.o., **C**), Aβ + **2** (250 mg/kg, i.p., **D**) Aβ + **2** (500 mg/kg, i.p., **E**). (**F**) Quantification of GFAP staining in the hippocampal region of all treatment groups. Data are shown as mean ± SEM. For comparisons between groups, a one-way ANOVA with Tukey’s post-hoc test was used for data analysis. ** *p* < 0.01, **** *p* < 0.001. Black arrows in panel B display typical GFAP positive staining of reactive astrocytes. All scale bars represent 25 μm.

The i.c.v. Aβ^1–42^ model replicates neuroinflammatory consequences of AD pathology [25]. The amyloid plaques in the AD brain are often associated with reactive astrocytes. Astrocytosis or astrocyte reactivity is one of the pathological processes that present spatial distribution similar to that of amyloid plaques and is correlated with the expression of proinflammatory cytokines such as TNF-α and IL-6 [33,34]. Glial fibrillary acidic protein (GFAP) presents as a marker to measure astrocytosis in the brain and mitigation of this marker could indicate beneficial effects against AD-related immunological changes [35]. We analyzed expression of GFAP in the hippocampal region of the brain given its important role in cognitive function (Figure 5). Baseline expression of GFAP was noticed in the saline treated control due to invasiveness of the i.c.v. method. A 64% increase in GFAP levels was observed in the Aβ^1–42^-only treated group (Figure 5B,F). Near complete amelioration of reactive astrocytes was observed in compound **2**-treated animals (Figure 5C–E). A marginally better trend was observed in oral treatment of **2** compared to i.p. administration. There is increasing evidence that oxidative stress and neuroinflammation are contributing factors toward pathogenesis and progression of AD [2,36,37]. Effective mitigation of oxidative and inflammatory phenomenon could be beneficial in preventing severity and cognitive consequences of AD pathology.

### 3.8. Supplementation of Glycine Improved Neuroprotection Offered by the Dipeptide of ψ-GSH in i.c.v. Aβ^1–42^ Treated Mice

**Figure 6:**
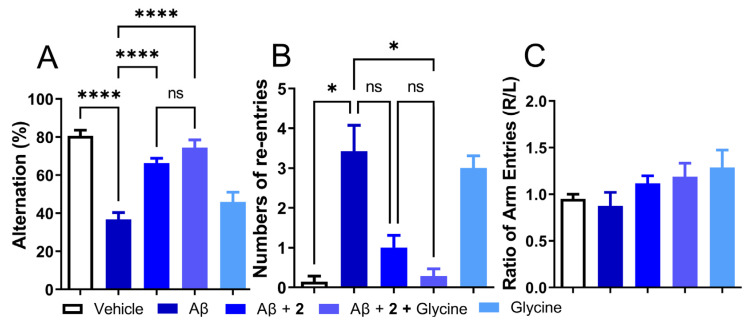
Co-treatment with ψ-GSH dipeptide (**2**, 250 mg/kg, i.p.) with glycine (250 mg/kg, i.p.) showed improved cognitive benefits over the treatment of compound **2** alone. (**A**) Impaired alternation in Aβ-injected mice was restored by compound **2** treatment and showed a trend toward further improvement in the co-treatment (**2** + glycine) group. (**B**) Increased number of re-entries in the Aβ-only treated group was significantly reduced in the co-treatment of compound **2** + glycine group, compared to treatment of compound **2** alone. (**C**) The treatment group did not show any side preference during the conduction of this test. Data are shown as the mean ± SEM. Statistical significance was assessed by a one-way ANOVA with Tukey’s post-hoc test (ns = not significant, * *p* < 0.05, **** *p* < 0.001).

Given the modest effect of compound **2** as an antioxidant by itself or by utilization of antioxidant enzymes and beneficial behavioral and biochemical effects in the i.c.v. Aβ^1–42^ model, the role of **2** as a precursor of ψ-GSH needs to be investigated further. From the enzymatic GSS assay, it is expected that the dipeptide compound **2** utilizes GSS in vivo to synthesize active tripeptide ψ-GSH. This pathway also requires non-essential amino acid glycine. Levels of glycine in glial cells and neurons are regulated by Na^+^/Cl^−^-dependent glycine transporters and metabolism to carbon dioxide and ammonia or conversion to serine by serine hydroxymethyl transferase (SHMT) [38]. It also plays a neurotransmitter role by modulation of the N-methyl-D-aspartate (NMDA) receptors. Beneficial effects of glycine have been observed in schizophrenia, metabolic disorders, obesity, and diabetes, to name a few [39]. We envisioned supplementing activity of compound **2** by provision of glycine, which in turn would result in higher levels of bioactive tripeptide ψ-GSH. When tested in the i.c.v. Aβ^1–42^ mouse model, glycine itself was ineffective in mitigating behavioral consequences of Aβ^1–42^ injection assessed by the T-maze test (Figure 6). When combined with compound **2**, the combination improved the alternation behavior displayed by compound **2** only group (74.49% for **2**+glycine vs 66.33% for Aβ^1–42^-only group, respectively; Figure 6A). Improvement in the number of repetitive arm entries reached statistical significance when compared to the Aβ^1−42^-only treated group (0.29 for **2**+glycine vs. 3.43 for Aβ^1–42^-only group, *p* < 0.05; Figure 6B). The results of this experiment highlight the importance of ψ-GSH formation in the neuroprotective activity offered by compound **2**.

## 4. Discussion

Supplementation of intracellular GSH levels using various formulations, precursors, or prodrugs has been a well-studied approach due to diverse physiological roles of GSH [40,41]. We studied an alternate approach to boost GSH concentrations by provision of a γ-GT-resistant GSH analogue, ψ-GSH. The compound is able to restore activities of various GSH-dependent enzymatic systems. In this study, we describe our attempt to utilize a precursor of ψ-GSH with the intent of increasing its accumulation at the target site of action. The dipeptide of ψ-GSH, compound **2**, retained the γ-GT-resistant ureide linkage and reduced thiol functional group present in ψ-GSH. This imparted innate antioxidant potential to **2**, along with ability to activate antioxidant enzyme glutathione peroxidase (GPx). The dipeptide **2** was able to form bioactive ψ-GSH tripeptide in vitro and in vivo. The compound was also confirmed as a substrate of glutathione synthetase (GSS) enzyme, one of key players of GSH biosynthetic pathway. These multifaceted activities of **2** lent neuroprotective activity against Aβ^1–42^ peptide in cell culture. When tested in an acute model of AD generated by i.c.v. injection of oligomeric Aβ^1–42^ solution, compound **2** was efficacious in restoring cognitive function and oxidative and neuroinflammatory consequences of amyloid pathology. Additional study in combination with glycine, the amino acid required for synthesis of tripeptide ψ-GSH from compound **2**, highlighted significance of tripeptide formation in the mechanism of action of **2**.

Alzheimer’ disease is the most prevalent cause of dementia amongst the elderly. Genetic factors, extracellular amyloid plaques, and neurofibrillary tau tangles being the presently known contributors, the multifaceted pathology of AD means that complete and accurate description of its pathophysiology is difficult, only to be underscored further by limited clinical success of therapeutics engaging a singular putative target [42]. For instance, antioxidant therapies were considered promising in preclinical studies; however, their successful clinical translation has been limited [12]. True disease-modifying treatments would likely necessitate targeting multiple aspects of the disease. Designed to restore glyoxalase enzyme function in the brain, antioxidant ψ-GSH presents additional benefits such as mitigation of oxidative stress and inflammatory pathology of AD. The compound was efficacious in transgenic mouse models of AD when administered in asymptomatic and symptomatic stages [22,23]. Thus, ψ-GSH presents a promising true disease-modifying option for AD. In an effort to improve permeability and oral availability of ψ-GSH, we sought to investigate precursors and prodrugs that would either deliver the active compound itself at pharmacologically relevant concentrations to the brain or would provide the missing link to the ravaged GSH biosynthetic machinery—effectively curing it. The precursor, compound **2**, can be viewed as a multifaceted address that will engage various biochemical pathways, directly or indirectly, whose compromise is what is presently understood as the observable pathophysiology of AD.

Apart from application of N-acetylcysteine (NAC) for the purpose of enhancing intracellular GSH levels, dipeptide precursors of GSH such as Cys-Gly and γ-Glu-Cys have also been extensively studied [43]. Dose-limiting toxicities of NAC, such as anaphylactic reactions, nausea, vomiting, are well known and are preceded by release of histamine [44]. It is true that chronic/long-term therapy with NAC is clinically employed, but the usual doses (600 mg PO BID) are far too small to cause a meaningful thiol augmentation in the brain. The dipeptide Cys-Gly can provide the needed amino acids for GSH biosynthesis due to its rapid catabolism but does not address the rate-limiting step in the biosynthetic pathway. The γ-Glu-Cys dipeptide is able to side-step the rate-limiting step in GSH synthesis catalyzed by γ-glutamylcysteine synthetase [45]. It could be directly incorporated into the final step by glutathione synthetase and circumvented feedback inhibition of γ-glutamylcysteine synthetase by GSH [43]. The γ-Glu-Cys dipeptide also displayed the ability to restore activity of GPx as a cofactor. However, the susceptibility of GSH to γ-GT-mediated breakdown is conserved in its dipeptide, γ-Glu-Cys. Compound **2** presents a similar advantage over γ-Glu-Cys dipeptide to that of ψ-GSH over GSH, by resisting γ-GT mediated cleavage.

Compound **2** bears a thiol essential for its antioxidant effect. In vivo pharmacokinetic evaluation confirmed it to be a liability, responsible for the short half-life of this compound. The significant metabolites that were detected in our in vitro stability studies included oxidized forms of the dipeptide and tripeptide ψ-GSH. We formulated strategies previously to address this issue in ψ-GSH itself by designing thiol-protected, orally available ψ-GSH prodrugs (manuscript under review). We will apply those strategies to compound **2** to augment its pharmacokinetic profile. Based on the therapeutic potential ψ-GSH revealed through our previous studies, supplementation of ψ-GSH using its precursors is a valid approach to alter the ongoing AD pathology and related biochemical changes, including progressive neurodegeneration. Given the multipronged approach presented by ψ-GSH dipeptide to tackle consequences of AD pathology, we are optimistic that further optimization and aggressive evaluation in transgenic AD animals would strongly support our approach and justify efforts for further clinical translation studies.

## 5. Conclusions

This account describes the conceptual genesis and experimental use of the dipeptide **2** as a precursor of established bioactive tripeptide ψ-GSH as a therapeutic for AD. The results indicate retention of antioxidant properties and successful activation of GSH-dependent enzymes by the dipeptide **2**, although less efficiently than that by ψ-GSH itself. Treatment with compound **2** delivered ψ-GSH, augmenting its own beneficial effects, and has also shown promise by restoring cytosolic pools of endogenous antioxidant GSH. In vivo beneficial effects of compound **2** were evident in the mitigation of hampered cognition and neuroinflammation as assessed by GFAP expression representative of astrocytosis in the i.c.v. Aβ^1–42^ mouse model. Collectively, this study suggests that the use of precursors of ψ-GSH that retain the γ-GT-resistant link as a valid approach for the development of therapeutics against AD. Limited stability of dipeptide **2**-based precursors due to the presence of labile sulfhydryl group warrants further attention and can be perhaps addressed through the design of thiol-protected prodrugs of such compounds.

## Figures and Tables

**Table 1:** Glutathione synthetase (GSS) assay.

Time (min)	GSH from γ-Glu-Cys (μM)		Ψ-GSH from Dipeptide 2 (μM)	
	(+)-GSS	(−)-GSS	(+)-GSS	(−)-GSS
0	7.9	ND ^1^	7.0	ND
5	113.1	ND	90.7	ND
15	428.5	ND	274.5	ND
30	917.8	ND	541.1	ND
60	1793.8	ND	1064.7	ND

ND ^1^ = not detected.

## Data Availability

Chemical compounds and all raw data or original images will be made available upon request made to More.

## Supplementary Materials

The following supporting information can be downloaded at: https://www.mdpi.com/article/10.3390/antiox11061075/s1, Figure S1: Spectral analysis of compound **2**; Figure S2: Measurement of intracellular ROS using DCFH-DA; Figure S3: Glutathione peroxidase enzymatic assay using ψ-GSH as a cofactor; Figure S4: Conversion of γ-Glu-Cys and compound **2** to GSH or ψ-GSH (**1**), respectively, after incubation in brain homogenates; Figure S5: Changes in body weights of mice treated in in vivo studies.
