# Supplementary material for: Dipeptide of ψ-GSH Inhibits Oxidative Stress and Neuroinflammation in an Alzheimer’s Disease Mouse Model
Source: Antioxidants (Basel). 2022 May 28;11(6):1075. doi: 10.3390/antiox11061075 (PMC9219802; doi:10.3390/antiox11061075)
Supplement: Supplementary file 1 [file antioxidants-11-01075-s001.zip › antioxidants-1737635-supplementary.pdf]

Supplementary File 1

Supplementary Figures S1-S5

For

Dipeptide of ψ-GSH Inhibits Oxidative Stress and Neuroinflammation in an Alzheimer's Disease Mouse Model

Abbas Raza [1,†], Wei Xie [1,†], Kwan Hyun Kim [1], Venkateshwara Rao Dronamraju [1], Jessica Williams [1], Robert Vince [1], and Swati S. More [1,*]

A

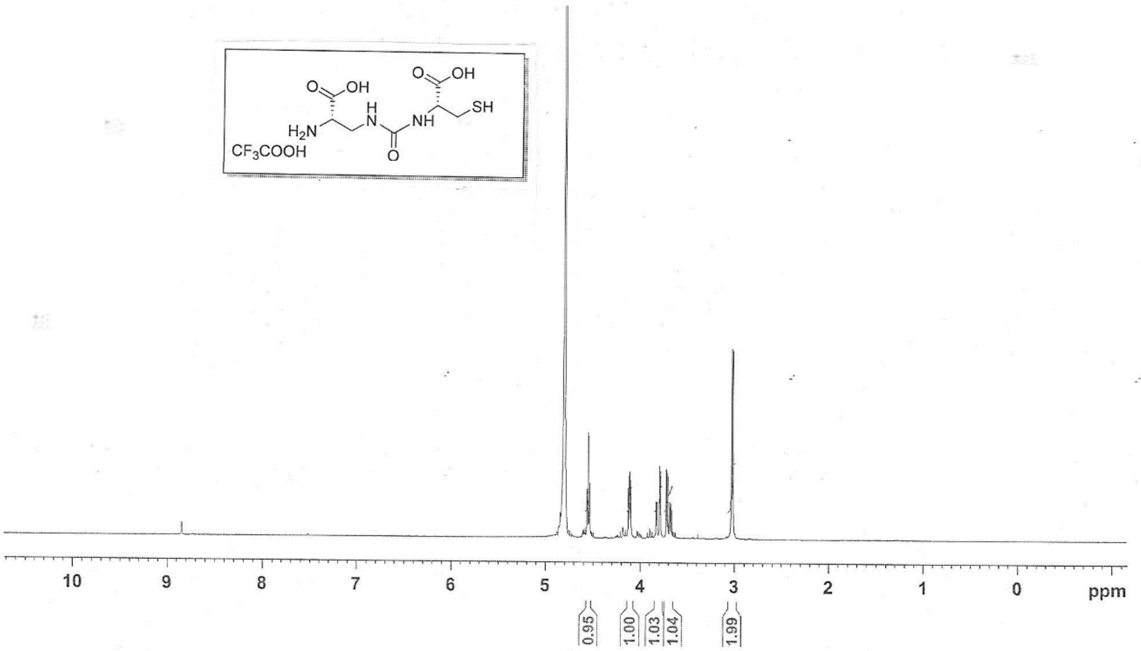

OH
H
N
NH
SH
O
H_2N
CF_3COOH
10
9
8
7
6
5
4
3
2
1
0
ppm
0.95
1.00
1.03
1.04
1.99

B

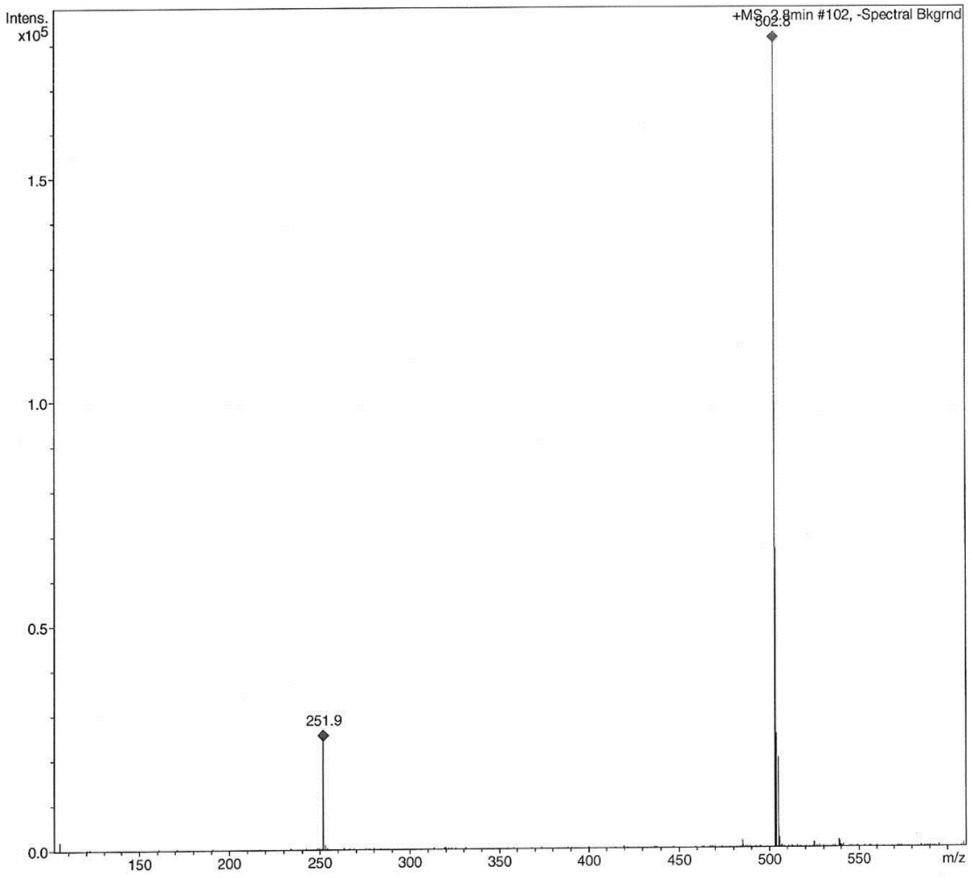

Intens.
x10^5
+MS, 2.8min #102, -Spectral Bkgrnd
1.5
1.0
0.5
0.0
251.9
150
200
250
300
350
400
450
500
550
m/z

C

Acq. Method : D:\METHODS\AB-5(15).M\AB-5(15).M
Analysis Method : D:\METHODS\AB-5(15).M\AB-5(15).M

Column:Synergi Fusion RP (150X4.6mm,4.0um)
Mobile Phase:0.1%Aq TFA
Flow Rate:1.0mL/min. Drift:60°c, Gain:5

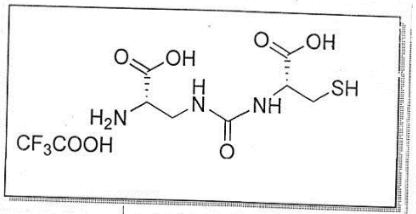

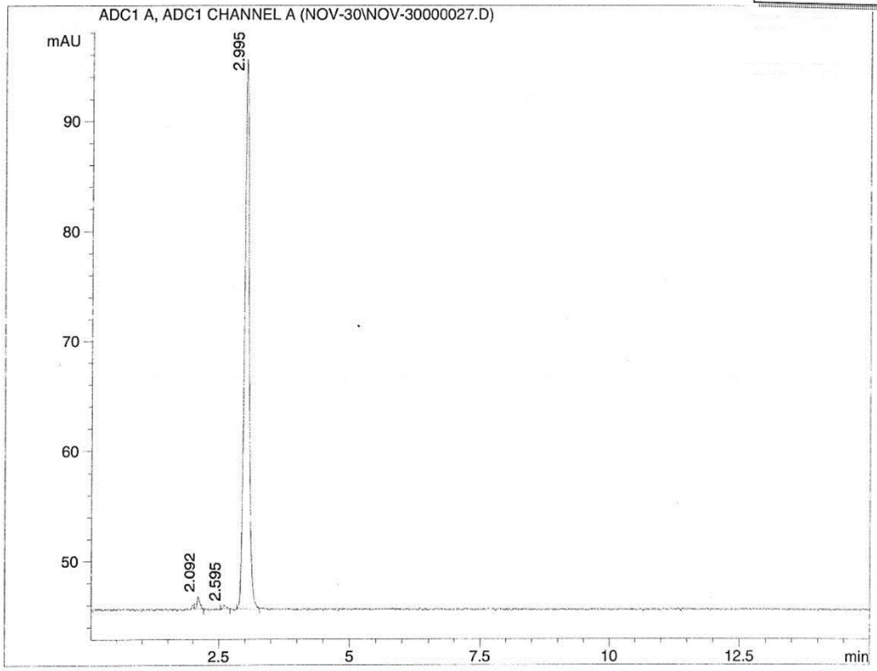

Signal 1: ADC1 A, ADC1 CHANNEL A

Peak #	RT [min]	Type	Width [min]	Area	Area %
1	2.092	MM	0.058	3.874	1.202
2	2.595	MM	0.066	1.322	0.410
3	2.995	MM	0.106	317.189	98.388

Figure S1. ^{1}H NMR (A), mass spectrum (B), and HPLC purity (C) analysis of compound **2**.

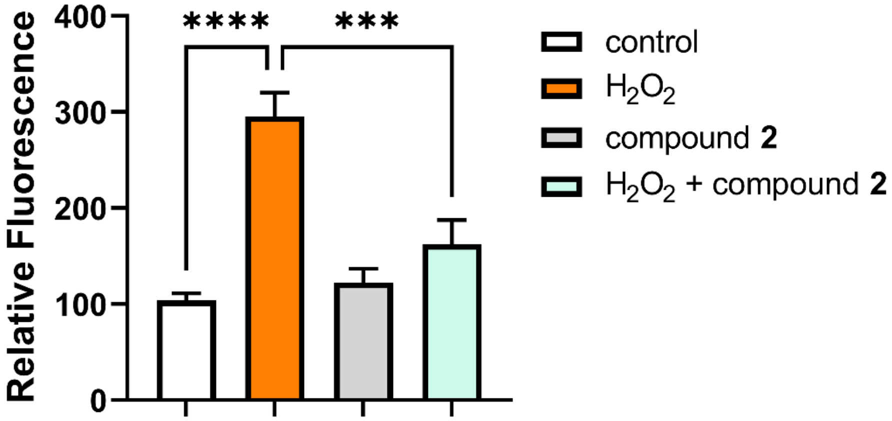

Figure S2. Measurement of intracellular ROS using DCFH-DA. Oxidative stress was induced in SH-SY-5Y cells by exposure to 500 μM concentration of H_2O_2 (500 μM) for 90 min. Treatment with compound **2** was initiated 30 min prior to H_2O_2 exposure and continued during the 90 min peroxide exposure period. Increase in DCF fluorescence was used as a measure of intracellular ROS content. The data are presented as the mean ± SEM of two independent experiments (*** *p* < 0.005, **** p < 0.001).

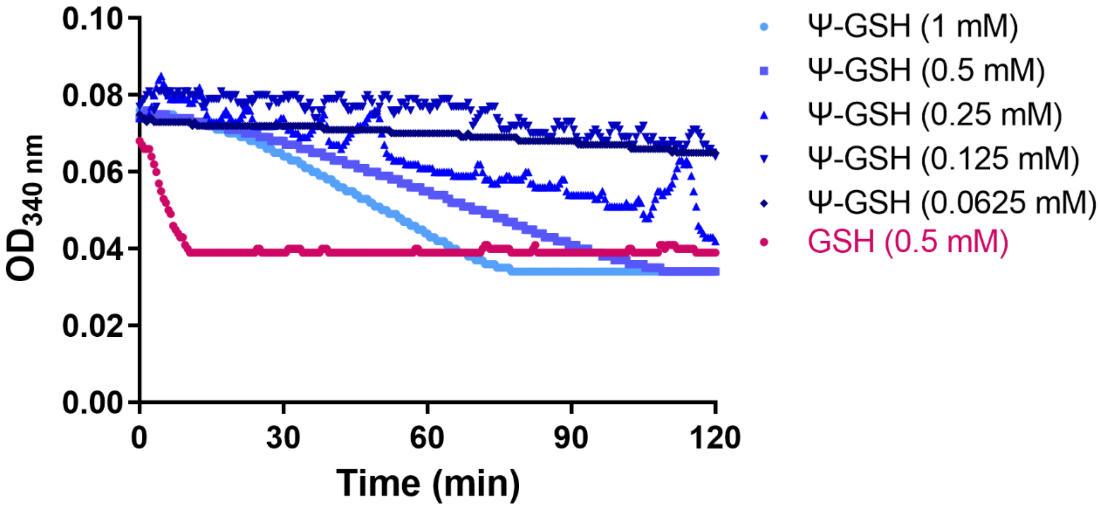

Figure S3. Glutathione peroxidase enzymatic assay using ψ-GSH as a cofactor. Measurement of glutathione peroxidase activity was performed using the purified enzyme in the presence of ψ-GSH or GSH as cofactors. In this coupled enzymatic assay, consumption of NADPH by glutathione reductase to reduce oxidized ψ-GSH or GSH was measured. Levels of NADPH measured as absorbance at 340 nm are plotted against time and used as an indicator of enzymatic activity.

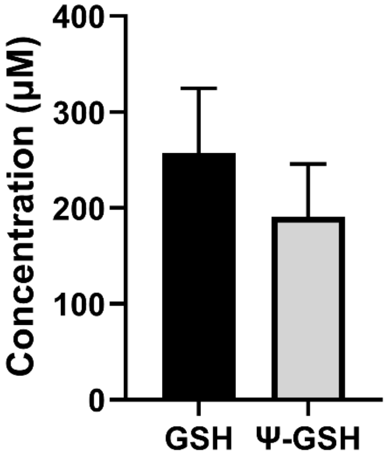

Figure S4. Conversion of γ-Glu-Cys and compound **2** to GSH or ψ-GSH (**1**), respectively, after incubation in brain homogenates. Compounds γ-Glu-Cys and **2** were incubated (final concentration 1 mM) at 37 °C for 2 hours and the levels of respective tripeptides formed were measured by LC-MS/MS analysis. Comparable formation of GSH and ψ-GSH were detected from respective tripeptides. Data are means ± SEM of two independent experiments.

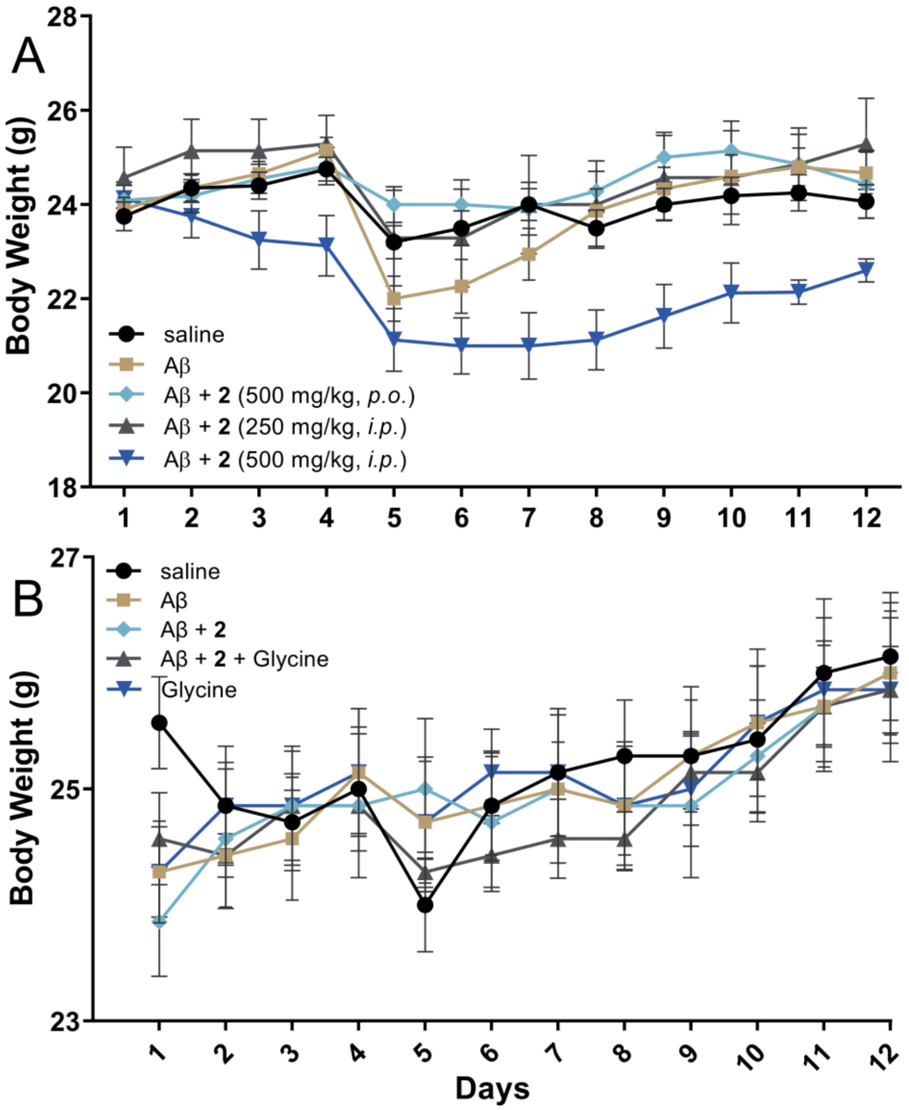

Figure S5. Changes in body weights of mice treated in studies related to sections 3.6 (A) and 3.8 (B). Except for the compound **2** (500 mg/kg, *i.p.*) group, none of the treatment groups showed any significant reduction in body weights.
